# Co-expression and impact of prostate specific membrane antigen and prostate specific antigen in prostatic pathologies

**DOI:** 10.1186/1756-9966-29-171

**Published:** 2010-12-28

**Authors:** Awatef Ben Jemaa, Yosra Bouraoui, Sataa Sallami, Ahmed Banasr, Nawfel Ben Rais, Latifa Ouertani, Yassin Nouira, Ali Horchani, Ridha Oueslati

**Affiliations:** 1 Unit of Immunology and Microbiology Environmental and Carcinogenesis (IMEC), Faculty of Sciences of Bizerte, 7021, Zarzouna, University of 7-November at Carthage, Tunisia; 2 Department of Urology, Hospital of La Rabta Tunis, Tunisia; 3 Department of Legal Medicine, Hospital of Charles Nicolle Tunis, Tunisia; 4 Department of Urology, Military Hospital of Tunis, Tunisia; 5 Department of Anathomopathology, Hospital of Menzel Bourguiba, Tunisia

## Abstract

**Background:**

The present study was undertaken to relate the co-expression of prostate-associated antigens, PSMA and PSA, with the degree of vascularization in normal and pathologic (hyperplasia and cancer) prostate tissues to elucidate their possible role in tumor progression.

**Methods:**

The study was carried out in 6 normal, 44 benign prostatic hyperplastic and 39 cancerous human prostates. Immunohistochemical analysis were performed using the monoclonal antibody CD34 to determine the angiogenic activity, and the monoclonal antibodies 3E6 and ER-PR8 to assess PSMA and PSA expression, respectively.

**Results:**

In our study we found that in normal prostate tissue, PSMA and PSA were equally expressed (3.7 ± 0.18 and 3.07 ± 0.11). A significant difference in their expression was see in hyperplastic and neoplastic prostates tissues (16.14 ± 0.17 and 30.72 ± 0.85, respectively) for PSMA and (34.39 ± 0.53 and 17.85 ± 1.21, respectively) for PSA. Study of prostate tumor profiles showed that the profile (PSA+, PSMA-) expression levels decreased between normal prostate, benign prostatic tissue and primary prostate cancer. In the other hand, the profile (PSA-, PSMA+) expression levels increased from normal to prostate tumor tissues. PSMA overexpression was associated with high intratumoral angiogenesis activity. By contrast, high PSA expression was associated with low angiogenesis activity.

**Conclusion:**

These data suggest that these markers are regulated differentially and the difference in their expression showed a correlation with malignant transformation. With regard to the duality PSMA-PSA, this implies the significance of their investigation together in normal and pathologic prostate tissues.

## Introduction

The prostate gland is the site of two most pathological processes among elderly men, benign prostatic hyperplasia (BPH) and prostate cancer (PC) [1]. According to the zonal origin, prostate cancer arising mainly in the peripheral zone (PZ), whereas the transition zone (TZ) is the exclusive location for the origin of BPH and PC developing in this latter zone are frequently found incidentally. There are different biological features between PZ and TZ of prostate gland [2]. Aberrant prostate growth arises as a consequence of changes in the balance between cell proliferation and cell death [3]. This deregulation may result in production of prostate specific markers such as the secreted protease prostate-specific antigen (PSA) and the cell surface prostate-specific membrane antigen (PSMA) [4]. A transmembrane glycoprotein expressed in the human prostate parenchyma, from where it was first cloned and named prostate-specific membrane antigen (PSMA) [5] has gained increased attention in diagnosis, monitoring and treatment of PC [6]. PSMA is a metallopeptidase belonging to the peptidase family M28 [7] and has apparent molecular masses of 84-100 kDa [8] with a unique three-part structure: a short cytoplasmic amino terminus that interacts with an actin filament, a single membrane-spanning domain and a large extracellular domain [9]. Several alternative isoforms have been described, including the cytosolic variants PSMA', PSM-C, PSM-D [10] and PSMA-E. These variants are thought to be the consequence of alternative splicing of the PSMA gene [11]. Concerning prostate tumorigenesis, the membrane form of PSMA is predominantly expressed. However, in normal prostate the dominating form of this protein is the one that appears in the cytoplasm [12,13]. If acting as a transmembrane receptor, PSMA can be internalized from the plasma membrane and trafficking through the endocytic system [13]. Although the PSMA have been noted in a subset of non prostatic tissues (small intestine, proximal renal tubule), the level of expression of PSMA in these tissues is less than in prostate tissue [14]. PSMA functions as folate hydrolase and neuropeptidase [15,16] with expression at low levels in benign prostatic epithelium and upregulated several fold in the majority of advanced prostatic malignancies [17]. In these tumors, PSMA immunoexpression has been shown to correlate with aggressiveness of the PC, with highest levels expressed in an androgen-deprived state and metastatic disease [18].

Unlike PSMA, PSA is a 33 kDa glycoprotein of the kallikrein family of proteases [19]. It is found in normal, hyperplastic and malignant prostate tissue, and is not specific biomarker for PC [20]. It is secreted into the lumen of prostatic duct to liquefy the seminal coagulum [21]. In invasive adenocarcinomas, disruption of the normal glandular architecture and loss of the polarity of prostatic cells appear to allow PSA increased direct leakage into peripheral circulation [22]. PSA is the most widely used serum marker for the diagnosis and follow-up of PC [23]. Unlike serum PSA, there are drawbacks to use tissue PSA, like for example, the loss of expression of tissue PSA associated with advanced disease and the development of androgen-independent prostate cancer (AIPC) [20,24].

Angiogenesis, the establishment of new blood vessels from preexisting blood, is thought to be required for process of tumorigenesis and metastasis and may prove to be a useful prognostic marker for prostate cancer [25]. A notable finding is that PSMA, an angiogenic endothelial cell which is like one of several peptidases that play a role in angiogenesis. PSMA expression was specifically detected on the neovasculature of many other prostates not related tumors, suggesting the possibility that PSMA may also functionally contribute to angiogenesis of primary and metastatic cancers [26,27].Therefore, it has been suggested that PSMA may be utilized both as a marker and as a therapeutic target [26,6].

In prostate cancer, a significant correlation between PSMA expression and angiogenesis has been shown [26,28]. However, the biological role of both angiogenesis [29] and PSMA expression in PC is still unclear for there are, indeed, studies in which the presence of these molecules is deprived of any prognostic significance [30].

Interestingly, *in vitro *and *in vivo *investigation, it was revealed that PSA suppresses angiogenesis and, therefore, tumor growth and PC invasiveness by activating the angiostatin-like fragments [31,32].

The present study was undertaken to relate the co-expression of prostate-associated antigens, PSMA and PSA, with the degree of vascularization in normal and pathologic (hyperplasia and cancer) prostate tissues to elucidate their possible role in tumor progression. On the basis of the heterogeneity in PSMA and PSA expression along prostatic tumor progression, we suggested the presence of various profiles of these prostate-associated antigens in each prostatic group (NP, BPH and PC). This led us to better investigate the association between the two markers in each prostatic group. The ultimate question was which, if any, of these factors could provide additional information regarding the biology of prostate tumorigenesis.

## Materials and methods

Prostates were obtained from: (i) transurethral resections from 44 men (aged from 61 to 85 years) diagnosed clinically and histopathologically with Benign Prostate Hyperplasia (BPH); (ii) radical prostatectomy from 39 men (aged from 57 to 90 years) diagnosed with prostate cancer (PC) (dominant Gleason grade ≥7); and (iii) histologically normal prostates (NP) obtained at autopsy (8-10 hours after death) from 6 men (aged from 21 to 40 years) without histories or reproductive, endocrine or related diseases.

All pathological, clinical and personal data were anonymized and separated from any personal identifiers. This study was made with the consent of the patients' relatives or their family in autopsy cases. All the procedures followed were examined and approved by the Hospital of La Rabta of Tunis, the Hospital of Charles Nicolle of Tunis and the Military Hospital of Tunis (HMPIT) (Tunisia).

The primary antibodies used were: mouse anti-human PSMA (3E6), mouse anti-human PSA (ER-PR8) and mouse anti-human CD34 (QBend10) (Dako, Glostrup, Denmark). CD34 antibody was used to label vessels in the prostate tissues.

For hematoxylin-eosin staining and immunohistochemistry analysis, tissues were fixed for 24 hours at room temperature in 0.1 M phosphate-buffered 10% formaldehyde, dehydrated and embedded in paraffin. Sections (3 mm thick) were processed following the NovoLink™Polymer Detection Systems (Novocastra Laboratories Ltd, Newcastle, UK) method. Sections were deparaffinized, rehydrated through graded alcohols and washed in de-ionized water. To retrieve antigens, sections were incubated in citric acid solution (0.1 M, pH 6) for 20 minutes in 98°C using a water bath. Slides were allowed to cool for another 20 min, followed by washing in de-ionized water. Endogenous peroxidase activity was quenched by incubation with Peroxidase Block for 5 minutes. Each incubation step was carried out at room temperature and was followed by two sequential washes (5 min each) in TBS. Sections were incubated with Protein Block for 5 minutes to prevent non-specific binding of the first antibody. Thereafter, the primary antibodies were applied at a dilution of 1/50 (PSMA) and 1/100 (PSA, CD34) in antibody diluents (Dako, Glostrup, Denmark) at room temperature for 30 minutes. Afterwards, the sections were incubated with Post Primary Block for 30 minutes to block non-specific polymer binding. The sections were incubated with NovoLink™Polymer for 30 minutes followed by incubations with 3, 3'-diaminobenzidine (DAB) working solution for 5 minutes to develop peroxidase activity. Slides were counterstained with hematoxylin and mounted. Stainig specificity was checked using negative controls. Prostatic tissues of each type were incubated in blocking peptides (Santa Cruz Biotechnology, Santa Cruz, CA, USA) instead of primary antibodies.

A comparative quantification of immunolabeling in all tissues types was performed for each of the three antibodies. Of each prostate, six histological sections were selected at random. In each section, the staining intensity (optical density) per unit surface area was measured with an automatic image analyzer (Motic Images Advanced version 3.2, Motic China Group Co., China) in 5 light microscopic fields per section, using the ×40 objective. Delimitation of surface areas was carried out manually using the mouse of the image analyzer. For each positive immunostained section, one negative control section (the following in a series of consecutive sections) was also used, and the optic density of this control section was taken away from that of the stained section. From the average values obtained (by the automatic image analyzer) for each prostate, the means ± SEM for each prostatic type (normal prostate, BPH and PC) were calculated. The number of sections examined was determined by successive approaches to obtain the minimum number required to reach the lowest SEM. The statistical significance between means of the different prostate group's samples was assessed by the Fisher exact test and the one-way ANOVA test at p≤0.05 (GraphPad PRISMA 5.0 computer program).

## Results

We examined human histological specimens (NP, BPH and PC) by immunohistochemistry to evaluate the relationship between the co-expression of prostate- associated antigens (PSMA and PSA) and the degree of vascularization (intensity of immunoreaction to CD34).

**Figure 1:**
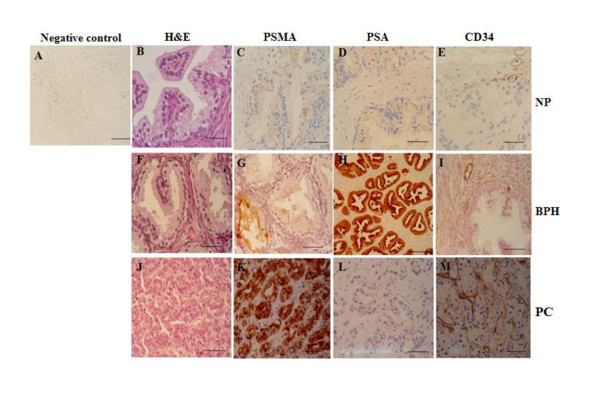
**H & E stained slides in NP (B), BPH (F) and PC (J)**; **immunohistochemical localizations of PSMA, PSA and CD34**. Negative control (A). NP showing weak cytoplasmic staining for PSMA (C) and PSA (D) in epithelial cells. CD34 was found at low level in membranous and cytoplasmic endothelial cells in NP (E) and BPH (I). BPH showing weak membranous staining for PSMA (G) and strong membranous and cytoplasmic staining for PSA (H) in prostatic epithelial cells. PSMA (K) and CD34 (M) showed strong immunoreactions in infiltrating prostatic carcinoma. PSA (L) showed weak cytoplasmic immunoreactions of epithelial cells in PC. Scale bars: A-G, I-M, 20 μm; H, 30 μm.

We didn't see any immunoreactivity in the negative controls incubated with blocking peptides (Figure 1A). Immunorectivity for PSMA appeared in 83% of NP, 86% of BPH and 97% of PC samples. In NP and BPH samples, PSMA was exclusively expressed in the cytoplasm of luminal epithelial cells, whereas we found it only expressed in the tumor cells of the PC specimens. We wanted to look at the expression of PSMA in blood vascular, we stained adjacent sections with anti-CD34 and anti-PSMA antibodies of our samples and we found that endothelium of both benign and malignant prostate tissues were deprived from PSMA expression (Figure 1C, G and 1K).

**Figure 2:**
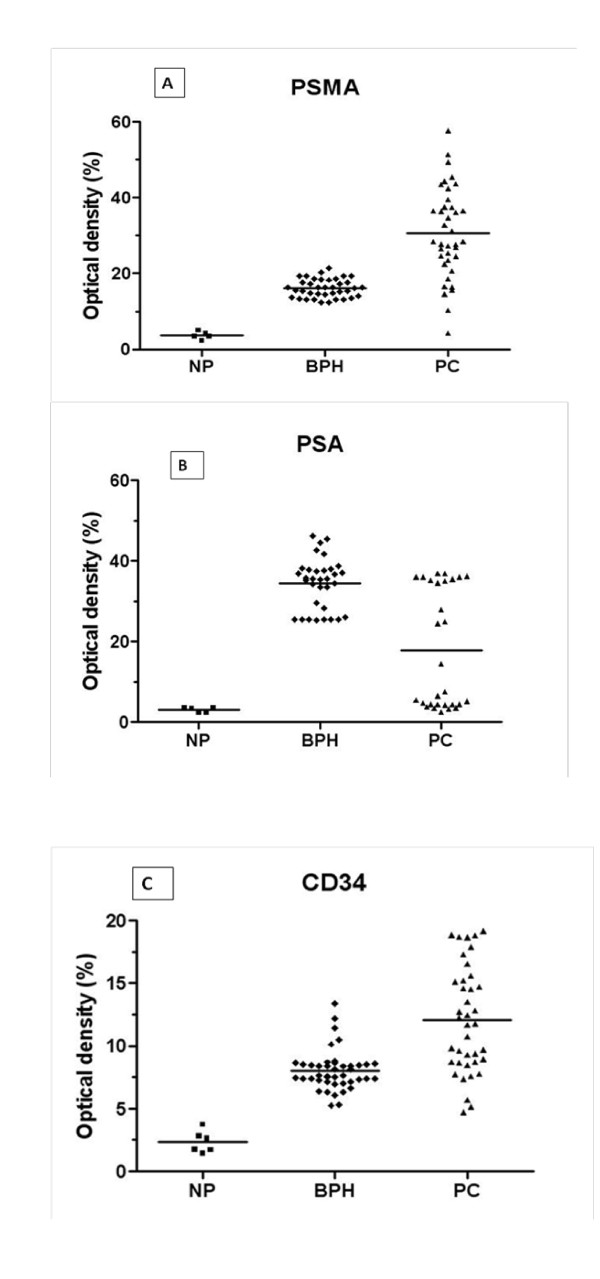
**Distribution of tissue PSMA (A), PSA (B) and CD34 (C) immunostaining intensity (measured as average optical density) according to normal prostate (NP), benign prostatic hyperplasia (BPH) and prostatic carcinoma (PC)**. Average optical densities were evaluated only in patients showing immunopositivity.

We used Motic advanced software to calculate the optic density (OD) that correlates with the antigen expression. We found that the mean of PSMA expression was significantly increased in benign prostate glands compared with normal prostate tissue (respectively 16.14 ± 0.17 and 3.7 ± 0.18) (p = 0.008). The highest level of PSMA expression was found in primary prostate cancer (30.72 ± 0.85) which significantly differed from benign (p < 0.0001) and normal prostatic tissue (p < 0.0001) (Figure 2A). Unlike PSMA, PSA expression was found the highest in hyperplastic epithelial cells (Figure 2B). Scanty immunoreactivity to PSA was localized in the cytoplasm of epithelial cells in normal prostate (Figure 1D). Figure 2B showed that the intensity of immunoreaction to PSA decreased from BPH samples to prostate adenocarcinoma (34.39 ± 0.53 and 17.85 ± 1.21, respectively) (p < 0.0001). As shown in this figure, 57% of PC samples positive for PSA have a similar PSA expression level distribution to NP samples, whereas 43% have a similar PSA expression level distribution to BPH samples. PSA staining was present in 83% of NP, 75% of BPH samples and 74% of PC samples.

To look at the vasculature in our samples, we immunostained them with anti-CD34 mouse using IHC method. CD34 consistently showed immunoreactivity in the plasma membrane of endothelial cells in all prostates specimens (Figure 1E, I and 1M). Measuring the optical density of CD34 immunostaining, we found that there is a significant difference in vasculature density between normal, hyperplasia and tumors in our collection (Figure 2C). Interestingly, similar to PSMA, CD34 staining was found more abundant in PC specimens (12.08 ± 0.29), compared with NP and BPH (p < 0.0001). Vessel density was higher in BPH compared to NP samples (8 ± 0.11 and 2.34 ± 0.15, respectively) (p < 0.0001) (Figure 2C).

**Figure 3:**
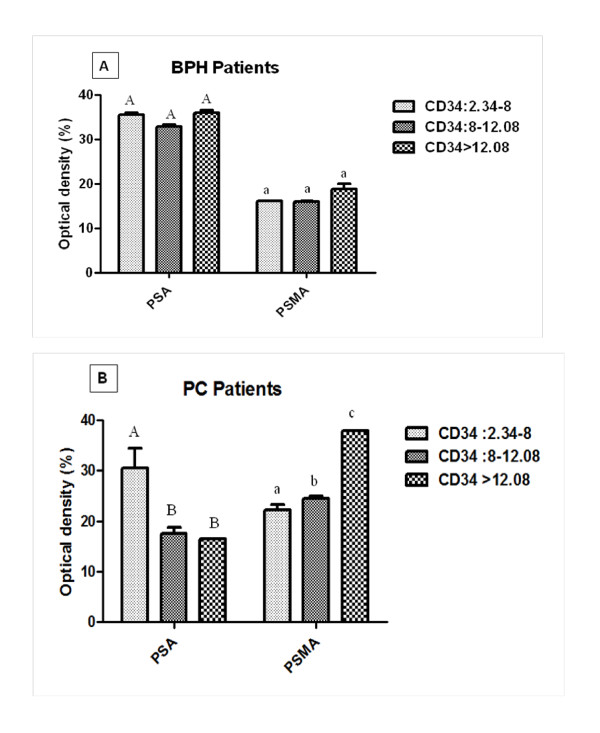
**Association between immunostaining intensity of CD34, PSMA and PSA expression among tissue CD34 levels in benign prostatic hyperplasia (BPH) (A) and prostate cancer (PC) patients (B)**. Values were expressed as mean ± SEM. Average optical densities were evaluated only in patients showing immunopositivity. Statistical analysis refers to each antibody separately. Values denoted by different superscripts are significantly different from each other. Those values sharing the same superscript are not statistically different from each other. Statistical analysis refers to each antibody separately. Significance was determined at p≤0. 05; 2.34: Mean O.D of CD34 value in NP; 8: Mean O.D of CD34 value in BPH and 12.08: Mean O.D of CD34 value in PC patients.

To study the relationship between PSMA and PSA expression and microvessel density in BPH and PC samples, we divided BPH and PC samples into 3 subgroups. The first group has a CD34 OD values between 2.34 and 8, the second group has a CD34 OD values between 8 and 12.08 and the third group has a CD34 OD value superior to 12.08 (Figure 2C and Figure 3).

In BPH samples, no difference neither in PSA nor PSMA expression was found in all 3 subgroups (Figure 3A).

Importantly, depending on the degree of vascularisation, we found an inverse relation between angiogenesis and PSA in PC patients. Unlike PSA, the highest intratumoral angiogenesis is accompanied by high PSMA expression in prostate cancer cells (Figure 3B).

**Figure 4:**
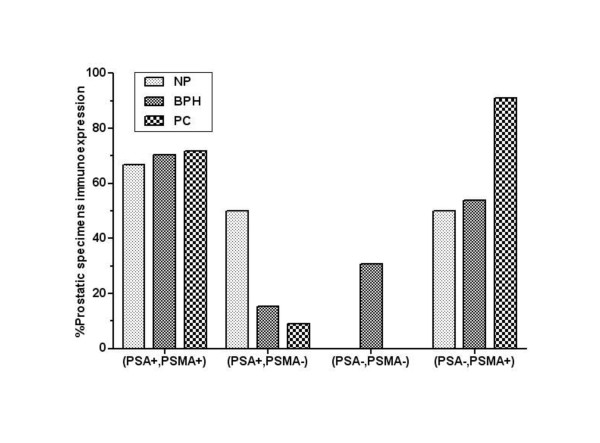
**Percentage of prostatic specimens with positive or negative immunoreactions to PSA and PSMA according to groups: normal prostate (NP), benign prostatic hyperplasia (BPH) and prostatic carcinoma (PC)**. Statistical analysis refers to each group separately at p≤0.05.

**Figure 5:**
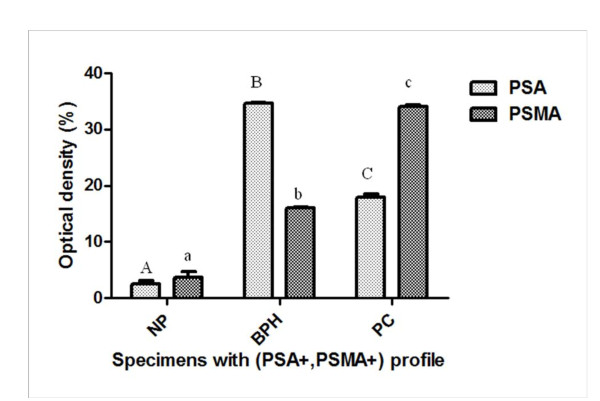
**Comparison of the intensity of immunoreactivity (measured as average optical density ± SEM) for PSA and PSMA according to groups: normal prostate (NP), benign prostatic hyperplasia (BPH) and prostatic carcinoma (PC) among (PSA+, PSMA+) profile**. Values denoted by different superscripts are significantly different from each other. Those values sharing the same superscript are not statistically different from each other. Statistical analysis refers to each antibody separately. Significance was determined at p≤0. 05.

To study the distinct pattern of proteins tumour profiles produced by prostate epithelial cells we established different prostate-associated antigens profiles depending on positive immunoreactions to PSA and PSMA in NP, BPH and PC samples. We obtained a negative group for PSA and/or PSMA in each prostate type. The distribution of this group was as followed: 2 in NP, 13 in BPH and 11 in PC patients. Figure 4 showed 4 prostate-associated antigen profiles expressed differently in NP, BPH and PC patients as followed: (PSA+, PSMA+), (PSA+, PSMA-), (PSA-, PSMA-) and (PSA-, PSMA+). For all histological specimens, the profile (PSA+, PSMA+) was the most expressed in 66% of NP, 70% of patients with BPH and 71% of PC patients. However, no significance was observed between the different groups of prostatic specimens according to the percentage of immunoexpression of the profile (PSA+, PSMA+). To obtain insights into the relationship between PSA and PSMA production in the subgroup (PSA+, PSMA+) along prostatic diseases, we analysed the intensities of immunoreactions to PSA and to PSMA in NP, BPH and PC patients for the above profile. As observed in Figure 5, optical density of PSA increases significantly from NP to BPH and declines in PC samples in the profile (PSA+, PSMA+) (p < 0.0001). However, the intensity of immunoreaction to PSMA increases significantly from NP to BPH and malignant prostate specimens (p < 0.0001) in the same profile.

The prostate tumour profile (PSA+, PSMA-) expression levels decreases from NP to benign prostatic tissue and primary prostate cancer (50% vs. 15% vs. 2%, respectively). Inversely, the profile (PSA-, PSMA+) expression increases from NP to BPH and PC patients (50% vs. 53% vs. 90%, respectively). Compared to BPH patients, the profile (PSA-, PSMA-) was absent in both NP and PC tissues. This profile was found in 30% of hyperplastic prostate tissues.

## Discussion

A variety of pathological processes lead to the loss of the normal prostate glandular architecture including benign prostatic hyperplasia and prostate cancer and its associated metastases. Aberrant prostate epithelial cells growth may result in direct production of prostate-associated antigens such as the secreted protease prostate-specific antigen (PSA) and the highly specific membrane antigen present in their plasma membrane, prostate-specific membrane antigen (PSMA) [4]. PSMA is an integral cell surface membrane protein which is highly specific to prostate gland [14]. Adenocarcinoma of the prostate, like many epithelial malignancies, initiates in the terminally differentiated secretory epithelial cells [33]. In the present study we demonstrated expression of PSMA within the cells of the prostatic secretory epithelium in normal, hyperplastic and malignant prostate specimens. We observed an increase of PSMA expression in prostate cancer. It' is seems to indicate a more extensive role of PSMA in prostate cancer. Low expression in normal tissue would suggest a limited role of PSMA in normal human prostate and low expression in benign prostate hyperplasia tissue may suggest a limited role of this protein in hyperplastic tissue [17,34]. Our finding is consistent with previous reports using immunohistochemistry and multiplex PCR reactions to demonstrate the association between PSMA and tumor progression [17,34,35]. A notable finding in our study revealed that in NP the expression of PSMA and PSA seems to be identical. However, PSMA expression in hyperplastic and neoplastic prostates tissues appears to be inversed to the PSA expression. Although PSMA is more expressed in malignant prostate than benign prostatic hyperplasia, PSA is highly expressed in hyperplastic tissues. This is in part, thought to be due to the differences observed in several biological features between peripheral and transition zone of the prostate gland [2]. Although, the majority of the glandular tissue in prostate is located in the peripheral zone, the PSA tissue is secreted at higher levels by benign prostate epithelium arising exclusively in the transition zone compared to prostate cancer developing mainly in peripheral zone [36,22]. The majority of our samples diagnosed with prostate cancer have a Gleason grade ≥7. However, regarding to PSA expression we observed a bi-modal distribution of expression of this marker in carcinomatous prostate samples. This is seems to be related to two mechanisms of growth of this prostate cancer tissue (data not shown). The study of distinct pattern of prostate tumor profiles produced by prostate epithelial cells depending on positive immunoreactions to PSA and PSMA showed a high immunoexpression of the profile (PSA+, PSMA+) in all histological prostate tissues. In this latter profile, PSA and PSMA are more expressed in BPH compared to NP. The PSMA was highest in neoplastic cells, whereas PSA was highest in benign cells in the same profile. For the profile (PSA+, PSMA-) expression levels decreases between normal prostate, benign prostatic tissue and primary prostate cancer. Inversely, the profile (PSA-, PSMA+) expression increases from NP, BPH to PC patients. Compared to BPH patients, the profile (PSA-, PSMA-) is absent in both normal and prostate cancer tissue. These data suggest that these markers are regulated differentially in their expression and this difference seems to increase with malignant transformation [34]. The preponderance of PSMA or PSA expression in each prostatic subgroup depends on the cellular context. The heterogeneity of PSMA versus PSA expression under the same sub-group of prostate-associated profiles is, in part, thought to be due to the effect of androgen, cytokines, growth factors receptors, adhesion molecules and many other membrane-generated signals that all share the ability to efficiently regulate PSMA and PSA gene expression [37,28,38]. Numerous studies indicates that in the secretory epithelial cells of prostate gland, both PSMA and PSA transcriptions are androgen-dependent [39,40]. The emergence of androgen-insensitive tumor cells arising as a consequence of an adaptation to androgen withdrawal or from pre-existing androgen-independent clone [33]. According to the androgen levels, PSMA and PSA are different in several ways. In a previous report Denmeade SR et al, have identified PSMA as a gene that was up-regulated in the more aggressive androgen independent prostate cancer cell line C4-2B compared to the androgen-dependent cell line LNCaP [41]. Recently, in *vitro *cell-based analysis of PSMA expression was found that both dihydrotestosterone and 1α, 25-dihydroxyvitamin D3 (1, 25-VD) are involved in regulation of this protein [39]. In human PC, the up-regulation of PSMA seems to be a late event in tumor progression as the increase was detected in hormone refractory tumors compared to normal and benign tissue. Authors have also indicate that PSMA is important in very advanced prostate cancer [17,42]. Unlike PSMA, a loss of expression of tissue PSA has been associated to advanced prostate cancer and to transition into hormone refractory tumor growth [32,20]. In addition, several experimental studies have shown that androgen-independent tumors are more angiogenic than androgen-dependent tumors [43]. Therefore, our finding suggests a possible cross talk between PSMA, PSA and intratumoral angiogenesis and its involvement in tumor growth and metastasis. This relation allowed us to classify the prostate specimens into groups according to the intensity of immunoreactions to CD34. In BPH patients, no differences were found on the intensities of immunoreactions to PSA or to PSMA regarding the levels of CD34. By contrast, in PC patients depending on the degree of vascularisation, it was found an inverse relation between angiogenesis and PSA. Unlike PSA, the highest intratumoral angiogenesis is accompanied by high PSMA expression in prostate cancer cells. This clearly argues for the view that endothelial cell PSMA expression may be connected with angiogenesis factors production which contribute to neoplastic cell proliferation, motility as well as its contribution to angiogenesis of primary and metastatic cancers [28]. This view is also in line with the study of Tsui P et al, reporting that PSMA expression seems to correlate with vascular endothelial growth factor (VEGF) which stimulates the directed growth of endothelial cells toward malignancies through the process of angiogenesis [44]. The function of PSMA in late prostate cancer is unknown, but its ability to remodel extracellular matrix by proteolytic cleavage might be important. Contrary to PSMA, the results of an *in vitro *investigation revealed that PSA, similar to angiostatin, are implicated in suppressing angiogenesis and, therefore, also prostate cancer development or invasiveness [31]. The vascular suppressive action of PSA could explain the low proliferation rate of tumor prostate growth and the low of angiogenesis process in malignant prostate [32]. In the study of Papadopoulous et al, it was found that high PSA expression is accompanied by low intratumoral angiogenesis in cancerous prostate epithelial cells [32]. The association between high PSA expression and low intratumoral angiogenesis seems to be consistent with our finding that prostate cancer expresses significantly less of tissue PSA than benign prostate tissue. The fundamental agent of angiogenesis, bFGF, promotes the proliferation and the migration of prostatic cancer cells by activation of MAPKs pathway and this effect of bFGF shows to be modulated by SOCS-3 (Suppressor of cytokine signalling-3)[28,45]. Interestingly, treatment with bFGF stimulates the expression of PSMA in LNCaP (androgen-dependent) cell line and restores the expression of this protein in disseminated form of prostate cancer, PC3 and DU145, (androgen-independent cells) [28]. Recently, Colombatti M et al, reporting for the first time a potential interaction of PSMA with signaling molecules by activating the NFkB transcription factor and MAPK pathways in prostate cancer LNCaP cell line. The authors suggested a possible cross talk between PSMA, IL-6 and RANTES chemokine and its implication in cell proliferation and cell survival in prostate cancer cells [37].

## Conclusion

In conclusion, these data provide further evidence that PSMA is an important factor in prostate cancer biology. Moreover, PSMA and PSA seem to be inversely regulated in prostate cells, especially in prostate cancer cells. Little information exists concerning the role of signaling pathway in regulating cell apoptosis and survival/angiogenesis in prostate cancer cells in context to PSMA and PSA co-expression, formed the basis of our future study. More understanding of their regulation within signaling cascade in our prostatic subgroups could be interesting.

## List of abbreviations

1, 25-VD: 1α, 25-dihydroxyvitamin D3; BPH: Benign prostate hyperplasia; NP: Normal prostate; O.D: Optical density; PC: Prostate cancer; PSA: Prostate specific antigen; PSMA: Prostate Specific Membrane Antigen; PZ: Peripheral zone; TZ: Transition zone.

## Competing interests

The authors declare that they have no competing interests.

## Authors' contributions

RO contributed to the conception and design of the study; RO and ABJ contributed to data analysis, interpretation and to manuscript writing; ABJ, YB, SS, AB, NBR, LO, YN and AH contributed to collection and assembly of data. All authors read and approved the final manuscript.
